# Chronic traumatic encephalopathy: identifying those at risk and understanding pathogenesis

**DOI:** 10.1007/s00415-017-8508-x

**Published:** 2017-05-17

**Authors:** M. D. Willis, N. P. Robertson

**Affiliations:** Institute of Psychological Medicine and Clinical Neurosciences, Cardiff University, University Hospital of Wales, Heath Park, Cardiff, CF14 4XN UK

Chronic traumatic encephalopathy (CTE) is a progressive neurodegenerative condition which can lead to dementia and is found in a proportion of individuals with a history of repetitive head injury. Clinical features are variable but include cognitive dysfunction, behavioural changes, Parkinsonism, and gait impairment. CTE has attracted considerable recent interest in both lay and medical literature in particular because of its association with a number of highly visible professional contact sports including American football, football, and rugby. As a result, sports regulatory bodies have had to ensure that updated protocols surrounding head injuries are strictly adhered to ensure player welfare. However, despite the link between head injuries and CTE, the exact mechanism remains poorly defined. To complicate matters further, there are currently no validated clinical diagnostic criteria for CTE, so that a reliable diagnosis can only be made by a post-mortem (PM) neuropathological examination. This inevitably restricts accuracy of prevalence estimates but most importantly is also likely to inhibit potential future therapeutic strategies directed towards at risk or pre-symptomatic individuals. An improved understanding of the mechanics of head injuries and how this might influence the risk of developing CTE would, therefore, be invaluable in providing insights into pathogenesis as well as predicting those at greatest risk.

In this month’s journal club, we present three papers, which attempt to answer some of these questions. In the first paper, the authors present a case series of retired footballers with dementia, examining the underlying pathology and highlighting the prevalence of CTE in this particular cohort of sports participants. The second paper attempts to identify cerebrospinal fluid (CSF) biomarkers associated with the presence of persistent symptoms of mild traumatic brain injury (mTBI). It is hoped that this will improve pathological understanding of the neurodegenerative processes triggered by mTBI and also identify individuals at risk of any associated neurodegenerative processes. The final paper offers a novel computational model to detail the location and extent of injuries produced by different mechanisms of head injury to understand the observed distribution of tau pathology in patients with CTE.

All these papers highlight the growing need for active, ongoing research into this important area of neurology. A clearer understanding more about the pathogenesis and risk factors for CTE will undoubtedly have wide ranging implications not only for contact sports but also for the wider population.

## Mixed pathologies including chronic traumatic encephalopathy account for dementia in retired association football (soccer) players

As widely publicised, CTE has been reported in a range of contact sports. Football is the most popular sport globally with head impacts with the ball and other players a common occurrence. Although many do not result in concussion, they can be associated with subtle neuropsychiatric deficits or changes in functional imaging—termed ‘subconcussion’. The authors of this paper identify repetitive head impacts as a potential cause of subclinical TBI and suggest that the risk of later development of CTE is a considerable public health interest. With only four previous reports of footballers with CTE, this study aimed to describe the clinical and pathological features of a group of retired professional footballers that developed dementia.

Between 1980 and 2010, 16 consecutive cases of retired footballers with progressive cognitive impairment were identified and followed-up until death. Fourteen cases consented to be included in the study and six to undergo PM examination. Collateral history, playing careers, and concussion history from close relatives were collected prospectively. All 14 were male with an average playing career of 26 years—13 were professional players and 1 a committed amateur. A single episode of concussion was reported in only six footballers. All developed progressive dementia in later life with an average age at onset of 63.6 years and disease duration of 10 years.

Following PM examination in six cases, fenestration of the septum was observed in all cases with cavum septi pellucidi in one case. All six demonstrated histological features supportive of CTE with four fulfilling diagnostic criteria. All had concomitant Alzheimer’s disease (AD) and TDP-43 pathology with cerebral amyloid angiopathy and hippocampal sclerosis also observed in five and two cases, respectively. Other concomitant diagnoses included corticobasal degeneration, Lewy body disease, and vascular disease. The authors speculate that the majority of cases in the ‘clinical’ groups (without PM examination) would also have mixed pathologies including CTE.

*Comment.* The authors hypothesise that CTE and probably AD and TDP-43 pathology in this cohort of retired footballers are related to their past prolonged exposure to repetitive subconcussive head impacts from heading and head-to-player collisions. Of interest, the rate of observed septal abnormalities was greater than the non-boxer general population, supporting a previous history of chronic repetitive head impacts. Although this study highlights an association between repetitive head impacts from playing football and the development of CTE, it is not possible to prove causal relationship. To advance understanding, it is likely that larger prospective studies with a variety of clinical outcomes will be required.

*Ling H et al. Acta Neuropathol. 2017;133*(*3*)*:337*–*352*

## Neurochemical aftermath of repetitive mild traumatic brain injury

Post-concussion syndrome (PCS) refers to patients whose symptoms attributable to mTBI persist for more than 3 months. At present, there are no objective biomarkers to assess central nervous system (CNS) damage in these patients. Furthermore, the relationship between PCS and future development of CTE is unknown. The main objective of this study was, therefore, to evaluate whether persistent symptoms of mTBI are associated with brain injury as determined by CSF biomarkers of CNS injury. This would allow improved characterisation of neurodegenerative processes triggered by mTBI and identify at risk individuals for PCS or progressive neurodegeneration. In particular, the authors hypothesised that PCS would be associated with increased CSF concentrations of the axonal proteins total tau (T-tau) and neurofilament light protein (NF-L) as well as the astroglial protein glial fibrillary acidic protein (GFAp). In addition, that there would be altered concentrations of Aβ1-42, phosphorylated tau (P-tau), and the synaptic biomarker Neurogranin (Ng).

This study included 16 male professional ice hockey players with PCS and 15 healthy controls in a multicentre cross-sectional study. All participants had normal imaging and underwent neuropsychological assessment with the Rivermead Post-Concussion Questionnaire (RPQ). CSF concentrations of the above biomarkers were measured in all participants.

Median time between most recent concussion and lumbar puncture was 4 months. Nine players had persistent PCS symptoms for more than 1 year. No differences were observed in NF-L concentrations between all PCS patients and controls, but when split into those with PCS for >1 year and those whose symptoms resolved within 1 year, increased NF-L concentrations were observed in the former group (*p* = 0.04). However, it should be noted that one of the control participants with a high concentration of NF-L was excluded from the analysis for reasons not fully explained. No differences in T-tau or GFAp concentrations were observed. With respect to biomarkers reflecting amyloid and tau pathology and synaptic loss, the PCS group had significantly (*p* = 0.05) lower levels of Aβ1-42 than the control group with no differences observed in p-tau or Ng. Higher RPQ scores were seen in the PCS group and correlated only with NF-L concentrations (*p* = 0.02). NF-L and p-tau both correlated with lifetime concussion events.

*Comment.* The authors conclude that increased CSF NF-L and reduced Aβ1-42 in patients with PCS are suggestive of axonal white matter injury and amyloid deposition. They suggest that measurement of these biomarkers may therefore be a future avenue to assess the degree of CNS injury in individuals with PCS and to distinguish individuals who are at risk of developing CTE. As the authors acknowledge, the main limitation of this study was the small sample size, and thus, larger cohorts will be required to confirm these findings as well as their relationship to other clinical and radiological outcomes.

*Shahim P et al. JAMA Neurol. 2016;73*(*11*)*:1308*–*1315*

## Computational modelling of traumatic brain injury predicts the location of chronic traumatic encephalopathy pathology

The pathology of CTE is characterised by intracellular deposits of hyperphosphorylated tau. The aggregation of this protein is particularly observed in the sulci, but the relationship to the biomechanics of injury and the development of pathology in these areas is unknown. This paper aimed to improve the understanding of this, so that future strategies such as improved helmet design could be implemented to ward against the long-term effects of TBI. To test this relationship, the authors developed a high fidelity 3D finite-element model of the human head, so that detailed investigation of brain deformation during impact loading could be measured by computer software. The authors hypothesised that head injury would lead to maximal deformation in the sulci.

Three different types of head injury were modelled, and strain (measure of maximum stretch within the element) and strain rates (measure of the maximum time rate of stretch within the element) were measured, both of which have been linked to neurodegeneration in in vivo and in vitro models. The three different scenarios included: a helmet-to-helmet impact in American football (reconstructed with dummies); an occipital head impact due to a fall from ground level (in silico reconstruction); and a road traffic accident (RTA) involving a helmeted motorcyclist (reconstructed at a purpose built helmet drop test facility). Once measured, the strain and strain rates as a result of these different reconstructions were loaded onto the computational model and results analysed. In a complementary analysis, diffusion tensor imaging (DTI) was performed in 97 TBI patients and 51 controls to investigate whether the site of neuropathology was found in areas predicted by the computational model.

Maximal strain and strain rates were the greatest in the sulci in the 3D finite-element model in both the American football and RTA cases. Maximal strain was observed in the sulci for the fall case but no significant differences for strain rate. A similar difference between sulcal and gyral changes was also seen when DTI data were examined from a group of TBI patients.

*Comment.* This study demonstrates that the predicted pattern of brain tissue deformation during head impact corresponds with the distribution of neuropathology reported in cases of CTE and corresponds with a sulcal location of diffusion MRI abnormalities in a large group of TBI patients. Using computational models, this study demonstrates that the nature of the head impact suffered by an individual can affect the pattern of brain injury parameters, which may influence the development of long-term neurodegeneration. These techniques could be used in the future to understand pathogenesis and to develop strategies to alleviate strain and strain rates in areas where it is greatest, such as in the design of new helmets.

*Ghajari M et al. Brain. 2017;140*(*Pt 2*)*:333*–*343*

